# Asthma: Smoking Clouds Treatment Benefits

**Published:** 2004-09

**Authors:** Ed Susman

Although there are numerous ways children with asthma and allergies can reduce attacks and live a more normal life, researchers at the 2004 annual meeting of the American Academy of Allergy, Asthma, and Immunology (AAAAI) in San Francisco said cigarette smoking in the home virtually negates those interventions. “The data are clearly there,” said Robert Holzhauer, a clinical assistant professor of pediatrics at the University of Rochester School of Medicine and Dentistry. “We have unequivocal data to show that sidestream smoke is dangerous to people with asthma.”

In one study presented at the March meeting, Holzhauer and colleagues identified Rochester schoolchildren aged 3–7 who had mild persistent to severe persistent asthma. Children were assigned randomly to school-based care groups. One group received daily inhaled corticosteroids—a proven, effective treatment to prevent asthma attacks—at school, while the other did not.

Children in the treated group had fewer attacks and school absences; their parents reported fewer worries about their children’s health, work absences, and unexpected changes in plans. But if there was smoking in the home, those advantages were almost completely nullified. These findings have since been published in the May 2004 issue of *Archives of Pediatrics and Adolescent Medicine*.

In another presentation, Dennis Ownby, chief of allergy and immunology at the Medical College of Georgia, described his examination of the relationship between exposure to cats and dogs during the first year of life and the risk of allergy at age 6–7 years. He selected a birth cohort of 474 children, who were classified as having no exposure to cats or dogs during the first year of life, exposure to 1 cat or dog, or exposure to 2 or more cats or dogs.

Children of nonsmoking parents were significantly less likely to have allergies if they were exposed to 2 or more cats or dogs; about 14% of these children were allergic, compared to 37.5% of children with 1 pet and 36.8% of children with no pets. But this benefit was not seen in children of smoking parents. “This research shows that cigarette smoking is not innocuous to young children,” Ownby said. “We see evidence that it’s affecting their immune system.”

Holzhauer said parents must be convinced of how important it is to stop smoking in the home if they have children with asthma. However the doctors stopped short of advocating persuasion through legislation. “I think that if we were to report these parents to the authorities for child abuse, we would lose the children as patients,” Holzhauer said.

Rather, Kathleen Sheerin, a private-practice allergy specialist and chair of the AAAAI Public Education Committee, suggested that pediatricians and other health care professionals more strongly emphasize to parents the link between asthma, allergies, and smoking. She said, “We counsel parents to go to another room to smoke or to go outside if there is a child in the house.”

